# Sacubitril/valsartan attenuates atrial conduction disturbance and electrophysiological heterogeneity with ameliorating fibrosis in mice

**DOI:** 10.3389/fcvm.2024.1341601

**Published:** 2024-01-19

**Authors:** Satoshi Iwamiya, Kensuke Ihara, Tetsushi Furukawa, Tetsuo Sasano

**Affiliations:** ^1^ Department of Cardiovascular Medicine, Tokyo Medical and Dental University, Tokyo, Japan; ^2^ Department of Bio-Informational Pharmacology, Medical Research Institute, Tokyo Medical and Dental University, Tokyo, Japan

**Keywords:** sacubitril/valsartan, neprilysin inhibitor, atrial fibrillation, optical mapping, electrophysiology, heterogeneity, C-type natriuretic peptide

## Abstract

**Background:**

Sacubitril/valsartan (SacVal) has been shown to improve the prognosis of heart failure; however, whether SacVal reduces the occurrence of atrial fibrillation (AF) in heart failure has not yet been elucidated. In this study, we aimed to determine whether SacVal is effective in reducing the occurrence of AF in heart failure and identify the underlying mechanism of its electrophysiological effect in mice.

**Methods:**

Adult male mice underwent transverse aortic constriction, followed by SacVal, valsartan, or vehicle treatment for two weeks. Electrophysiological study (EPS) and optical mapping were performed to assess the susceptibility to AF and the atrial conduction properties, and fibrosis was investigated using heart tissue and isolated cardiac fibroblasts (CFs).

**Results:**

EPS analysis revealed that AF was significantly less inducible in SacVal-treated mice than in vehicle-treated mice. Optical mapping of the atrium showed that SacVal-treated and valsartan-treated mice restored the prolonged action potential duration (APD); however, only SacVal-treated mice showed the restoration of decreased conduction velocity (CV) compared to vehicle-treated mice. In addition, the electrophysiological distribution analysis demonstrated that heterogeneous electrophysiological properties were rate-dependent and increased heterogeneity was closely related to the susceptibility to AF. SacVal attenuated the increased heterogeneity of CV at short pacing cycle length in atria, whereas Val could not. Histological and molecular evaluation showed that SacVal exerted the anti-fibrotic effect on the atria. An *in vitro* study of CFs treated with natriuretic peptides and LBQ657, the metabolite and active form of sacubitril, revealed that C-type natriuretic peptide (CNP) combined with LBQ657 had an additional anti-fibrotic effect on CFs.

**Conclusions:**

Our results demonstrated that SacVal can improve the conduction disturbance and heterogeneity through the attenuation of fibrosis in murine atria and reduce the susceptibility of AF in heart failure with pressure overload, which might be attributed to the enhanced function of CNP.

## 1 Introduction

Atrial fibrillation (AF), which is the most common type of cardiac arrhythmia, contributes substantially to a variety of potential complications such as stroke and heart failure (1, 2). In particular, the new-onset of AF during heart failure is strongly associated with an increased risk of mortality; therefore, the prevention of AF in patients with heart failure is in great demand (3, 4). According to previous studies, some anti-arrhythmic drugs and conceivable upstream therapies for AF have considerable risks and limited efficacy (5, 6). The angiotensin receptor-neprilysin (NEP) inhibitor, sacubitril/valsartan (SacVal), could be a possible drug for an upstream therapy based on a concurrent blockade of the renin-angiotensin-aldosterone system and NEP. NEP is a metalloendopeptidase that hydrolyzes and degrades multiple vasoactive substrates, including natriuretic peptides (NPs) and angiotensin II. The inhibition of NEP augments the potency of these substrates, which may result in favorable or unfavorable impacts on cardiac and vascular homeostasis (7, 8).

SacVal has been shown to exert a favorable influence on ventricular remodeling and improve clinical outcomes, including cardiovascular death and hospitalization, in patients with heart failure with reduced ejection fraction (HFrEF) and even in partial groups of heart failure with preserved ejection fraction compared with angiotensin-converting enzyme inhibitors (ACEi) or angiotensin receptor blockers (ARB) (9, 10). SacVal also exhibits enhanced efficacy in the reduction of ventricular arrhythmia, thus resulting in the decline of sudden cardiac death (11). This anti-arrhythmic effect of SacVal may be advantageous for heart failure management. Nevertheless, to date, most previous reports have affirmed its benefits exclusively for ventricles, and the anti-arrhythmic impact on AF remains unclear. Several meta-analyses have evaluated the efficacy of SacVal in the occurrence of AF compared to ACEi or ARB; however, they failed to prove SacVal's efficacy on AF (12, 13). The difficulty was attributed to several reasons: (1) these trials were not designed to investigate the impact of SacVal on the occurrence of AF, and (2) the precise detection of paroxysmal AF was difficult during follow-up. On the contrary, several cohort studies have reported the efficacy of SacVal in AF. One report demonstrated that SacVal treatment was associated with a lower risk of progression from paroxysmal AF to permanent AF than ARB therapy (14). Another study reported that SacVal decreases AF recurrence after catheter ablation in patients with AF (15). In addition, treatment with SacVal in patients with HFrEF after the implantation of implantable cardioverter defibrillators resulted in a remarkable reduction in AF occurrence (16). However, the precise molecular mechanism by which SacVal affects AF in failed hearts remains unclear.

In this study, we hypothesized that SacVal has favorable effects on atrial arrhythmogenicity accompanied by atrial remodeling in heart failure and then examined the anti-arrhythmogenic effect of SacVal on the atrium in mice.

## 2 Materials and methods

### 2.1 Mice and TAC operation

All animal experiments were performed in accordance with the Guidelines for the Care and Use of Laboratory Animals published by the National Research Council (The National Academy Presses, eighth edition, 2011), pre-approved and performed under the regulations of the Institutional Animal Care and Use Committee of Tokyo Medical and Dental University (Approval #A2017-024C3 and #A2022-175C3). Wild-type (WT) mice (C57BL/6jjcl) were purchased from CLEA Japan, Inc. All experiments were performed on male mice. For the *in vivo* study, C57BL/6jjcl mice aged 9–11 weeks (20–25 g of body weight) underwent transverse aortic constriction (TAC) or sham surgery as previously described (17). TAC operation was performed using 7-0 polypropylene with a 27-gauge needle as an indicator under anesthesia and mechanical ventilation. C57BL/6jjcl mice were prepared separately for the isolation of cardiac fibroblasts (CFs).

### 2.2 Drug administration

TAC-operated mice were divided into three groups to receive valsartan (Val; 30 mg/kg body weight) (Tokyo Chemical Industry Co., Tokyo, Japan) or sacubitril/valsartan (SacVal; 60 mg/kg body weight) (Angene Chemical, London, UK) dissolved in corn oil or just corn oil (vehicle; Veh). The dosages of Val and SacVal were selected according to previous studies (18–20). Corn oil was also administered to the mice in the sham-operated mice. Oral administration was performed by gavage twice daily starting the day after TAC or sham surgery. Drug administration was continued until the heart extraction was performed 14 days after surgery.

### 2.3 *In vivo* physiological measurements: plethysmograph, electrocardiogram, and echocardiogram

Blood pressure in awake mice was evaluated using a tail-cuff plethysmograph (BP-98AL; Softron, Tokyo, Japan), following the manufacturer's instructions. The mice were examined by plethysmography before surgery and on days 1, 7, and 14 after surgery. Surface electrocardiography (ECG) and ultrasound echocardiography (UCG) were performed in a blinded manner on postoperative day 14 as described previously (21, 22). During the examination, mice were anesthetized with less than 1.0% of isoflurane. The body temperature of mice was monitored during the experiments and maintained at 37.0°C using a heating pad and a light bulb.

### 2.4 *In vivo* electrophysiological study

Programmed stimulation for inducing AF, *in vivo*, was conducted using a custom-made pacing electrode inserted into the murine esophagus under anesthesia with 1.5% isoflurane. Body temperature was monitored and maintained at 37.0°C as described above. A pacing bipolar catheter was inserted into the esophagus, just dorsal to the left atrium, where the smallest capture threshold was obtained. All programmed electrical stimulations were delivered at a voltage that was twice the capture threshold. Atrial stimulus was performed with programmed stimulation up to triple extra stimulus pacing. Extra-stimuli were delivered after 10 atrial beats (S1) with a pacing cycle length (PCL) of 80 ms. The first extra stimulus (S2) was initially set 40 ms after the last pacing stimulus of S1. The S2 stimulus was delivered at progressively shorter coupling intervals with 5 ms steps until the effective refractory period (ERP). A second extra stimulus (S3) was added 40 ms after S2 and scanned with a 5 ms decremental step until the ERP was reached. A third extra stimulus (S4) was introduced after S3 in the same way as S2 and S3.

AF was defined as rapid and irregular beats in which the *P* wave was not recognized on the ECG and lasted for more than one second. The AF duration was defined as the interval between the initiation of arrhythmic atrial beats and the onset of the first recovered sinus beat.

### 2.5 Optical mapping

Optical mapping was performed in a blinded manner, with minor modifications, as described previously (23). The mice were intraperitoneally injected with unfractionated heparin (200 IU) and sacrificed by cervical dislocation. Hearts were rapidly excised and connected to a Langendorff perfusion circuit. Subsequently, the hearts were stained with 5.0 *µ*M of di-4ANEPPS (Santa Cruz Biotechnology, Dallas, Texas, USA), followed by the administration of 250 *µ*M of blebbistatin (Toronto Research Chemicals, Toronto, Canada). The fluorescence was recorded by Complementary Metal Oxide Semiconductor camera (MiCAM ULTIMA; Brainvision Inc., Tokyo, Japan) with a sampling rate of 0.2 ms and spatial resolution of 66 *µ*m. An *ex vivo* electrophysiological study was performed using a custom-made pacing electrode placed on the right atrium. The pacing stimulations were delivered at a voltage twice the capture threshold. Constant pacing was delivered with a pacing interval of 120 ms. The pacing interval was decreased in 20 ms steps until the cessation of 1:1 conduction. Action potential duration (APD) was obtained at 90% repolarization (APD_90_) for each image pixel. Conduction velocity (CV) was derived using an isochronal map as previously described (24). Action potential (AP) rise time was defined as the interval from the onset, which was at the highest second derivative of the AP, to peak AP deflection. The dependence of APD_90_, CV, and AP rise time on the PCL was also evaluated, namely rate adaptation. Heterogeneity was assessed using the coefficient of variation (CoV) from a map [defined as the standard deviation divided by the median value inside a region of interest (ROI)]. In all the above analyses, the ROI was commonly set inside the left atrium (LA), excluding the border. Comparisons between the groups were performed using the median values in the ROI on each map.

### 2.6 Histological assessment

The excised hearts were immersed in 10% buffered formalin and the paraffin sections were subjected to Masson's trichrome staining. The stained sections were photographed (DZ-710, Keyence, Osaka, Japan), and the fibrotic areas were quantified by using the ImageJ software (NIH). The proportion of fibrotic area was calculated as the ratio of the fibrotic area to the total cross-sectional area.

### 2.7 RNA extraction from the heart tissue and cultured cells, and quantitative PCR

Total RNA was extracted from heart tissue using the RNeasy Mini Kit (QIAGEN, Hilden, Germany) according to the manufacturer's protocol. cDNAs were synthesized using High-Capacity cDNA Reverse Transcription Kit (Thermo Fisher Scientific, Waltham, Massachusetts, USA) from 200 ng total RNA obtained from the heart tissue.

For RNA extraction from the cultured cells and cDNA synthesis, cell lysis and reverse transcription were conducted using the SuperPrep II Cell Lysis & RT Kit for qPCR (TOYOBO, Osaka, Japan) according to the manufacturer's instruction.

Quantitative polymerase chain reaction (qPCR) was performed using StepOnePlus (Thermo Fisher Scientific) with PowerSYBR Green Master Mix (Thermo Fisher Scientific) and custom-made primers. Expression levels were normalized to *Gapdh*. The primer sequences are listed in Supplementary Table S1.

### 2.8 Western blotting for atrial protein expression

Protein samples from murine atrial tissues were extracted using radioimmunoprecipitation assay buffer. Protein concentration was measured using a BCA protein Assay Reagent Kit (Thermo Fisher Scientific). Of the protein samples, 20 *µ*g was loaded on a 4%–15% SDS-PAGE gel (Bio-Rad Laboratories, inc., Hercules, California, USA) for the separation and transferred to PVDF membranes with Trans-Blot Turbo system (Bio-Rad Laboratories, inc.). After blocking with Blocking One or Blocking One-P (Nacalai tesque, Kyoto, Japan), the membranes were incubated overnight at 4°C with the primary antibody, as described in Supplementary Table S2. HRP-labeled secondary antibodies (Supplementary Table S2) were used to detect specific bands. Chemiluminescent signals were detected using an iBright CL1500 (Thermo Fisher Scientific). The band intensity was quantified using ImageJ software and normalized to that of GAPDH.

### 2.9 Isolation of adult primary cardiac fibroblasts and *in vitro* assays

Adult cardiac fibroblasts (CFs) were isolated and cultured as previously described with minor modifications (25). In brief, C57BL/6jjcl mice without surgery were sacrificed by cervical dislocation, and hearts were quickly excised, and CFs were isolated from whole murine hearts. The isolated CFs were resuspended with Dulbecco's Modified Eagle Medium/F12 (Nacalai tesque) with 10% FBS, followed by plating into 100 mm dishes and culture in a humidified chamber at 37°C with 5.0% CO_2_. CFs were used for *in vitro* assays between passage 1 and 3. After CFs were seeded in 96-well plates (3.0 × 10^4^ cells per well) and cultured for 24 h, culture medium was replaced with serum-free medium. At the same time, a pre-treatment with Atrial Natriuretic Peptide (ANP) (Phoenix Pharmaceuticals, Inc., Burlingame, California, USA), B-type Natriuretic Peptide (BNP) (Bachem, Bubendorf, Switzerland) or C-type Natriuretic Peptide (CNP) (MedChemExpress, Monmouth Junction, New Jersey, USA) at 1.0 or 5.0 *µ*M was initiated with or without antagonists of their cognate receptors; A71915 (Sigma Aldrich, Burlington, Vermont, USA) antagonizing natriuretic peptide receptor-A (NPR-A); P19 (Phoenix Pharmaceuticals, Inc.) antagonizing natriuretic peptide receptor-B (NPR-B); AP811 (R&D Systems, Minneapolis, Minnesota, USA) antagonizing natriuretic peptide receptor-C (NPR-C), at 1.0 *µ*M each, as well as LBQ657 (sacubitrilat; Cayman Chemical, Ann Arbor, Michigan, USA) at 10 or 50 *µ*M. Transforming growth factor β (TGFβ; PeproTech, Cranbury, New Jersey, USA) (1.0 ng/ml) was added 30 min after the application of NPs, followed by another incubation for 24 h. After incubation with the drugs, RNA was extracted from the cultured cells and reverse transcription was performed as described above. The mRNA expression levels of several fibrosis-related genes were assessed *in vitro*. LBQ657 was dissolved in dimethyl sulfoxide (DMSO), TGFβ in citric acid, and the other drugs in ultra-pure water. We established a vehicle control to which an equivalent dose of these solvents was added. The final concentrations of DMSO and citric acid were adjusted to be less than 0.1%.

### 2.10 Statistical analysis

All data are presented as mean ± standard error of the mean (SEM). Statistical analyses were performed using PRISM 9 software (GraphPad). Statistical differences were assessed using one-way or two-way ANOVA for multiple comparisons of individual means, followed by Tukey's post-hoc analysis. For the statistical comparison of AF inducibility, chi-square test adjusted using the Benjamini-Hochberg procedure was performed. *P* < 0.05 was considered statistically significant.

## 3 Results

### 3.1 SacVal ameliorated the impaired cardiac function and heart failure induced by pressure overload

**Figure 1:**
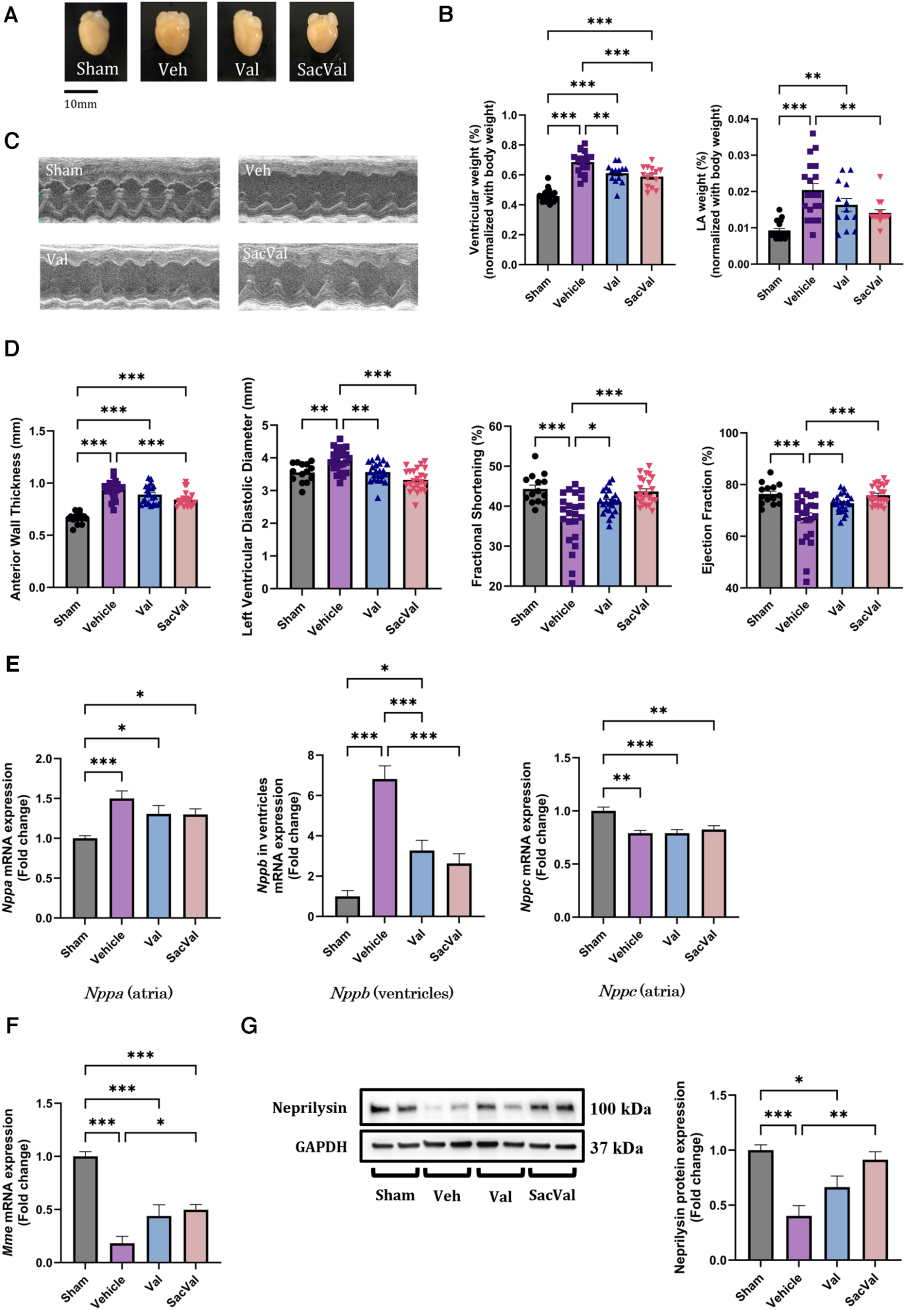
Physiological and molecular assessments of murine hearts with pressure overload showed the beneficial effects of SacVal. (**A**) Representative whole images of murine hearts underwent with sham surgery, TAC surgery (Vehicle; Veh), TAC surgery followed by valsartan (Val) or sacubitril/valsartan (SacVal) treatment. (**B**) Ventricular weight (left) and left atrial (LA) weight (right) normalized with body weight (*n* = 13–21). (**C**) Representative UCG images of M-mode. (**D**) Measured UCG parameters are shown from left to right, anterior wall thickness, left ventricular diastolic diameter, fractional shortening, and ejection fraction (*n* = 14–23). (**E**) Gene expression of *Nppa* (in atria), *Nppb* (in ventricles) and *Nppc* (in atria) (*n* = 6). (**F,G**) Gene and protein expression of *Mme* (encoding neprilysin) (*n* = 6). Multiple comparison was performed using one-way ANOVA with Tuckey's post-hoc test. Error bars, SEM. *P < 0.05; **P < 0.01; ***P < 0.001. TAC, transverse aortic constriction; UCG, ultrasound echocardiography.

Perpetual ventricular pressure overload by TAC is known to induce not only ventricular remodeling but also atrial remodeling, which is associated with the high inducibility of AF (26, 27). To establish the AF mouse model, we performed TAC surgery on WT mice, and acute pressure overload with TAC successfully caused heart failure with cardiac hypertrophy and deteriorated ventricular function in two weeks (Figures 1A,B). Regarding hypertrophy at the gene level, enhanced *Myh7* and reduced *Myh6* expression were observed in the ventricles with pressure overload, which was restored in the SacVal-treated group (Supplementary Figure S1). UCG showed that SacVal ameliorated the increase in wall thickness, dilation of the left ventricular diastolic diameter, and a significant decline in both fractional shortening and ejection fraction in comparison with the Veh (Figures 1C,D). Blood pressure was measured on days 1, 7, and 14 days after TAC procedure (Supplementary Figure S2A). Veh-, Val-, and SacVal-treated mice showed similar changes in peripheral blood pressure (Supplementary Figure S2B). The heart rate of mice in awake on postoperative day 14 did not differ between the groups (Supplementary Figure S2C). Furthermore, we found that the increase in left atrial weight in response to TAC was alleviated by SacVal and Val (Figure 1B). We measured the expression of the mRNA encoding ANP, BNP, and CNP: *Nppa, Nppb,* and *Nppc* respectively. TAC mice showed a significant increase in the expression of *Nppa* in atria and *Nppb* in ventricles. In contrast, the expression of *Nppc* in atria significantly decreased when exposed to pressure overload. Administration of SacVal significantly reduced the expression of *Nppb* in the ventricles as much as Val did, compared to the Veh group. In contrast, moderate reduction in the SacVal-treated group compared with the Veh-treated group was seen in that of *Nppa* in atria, and little change between them was observed in that of *Nppc* in atria (Figure 1E). Regarding ANP and CNP expression at the protein level in atria, ANP displayed similar trends to the gene expression of *Nppa*, but CNP showed different trends from its gene expression, indicating that the tissue concentration of CNP in atria increased under heart failure and SacVal modified the levels (Supplementary Figures S3A,B). Alternatively, the decreased gene expression of the cognate receptors of the NPs (*Npr1*, *Npr2*, and *Npr3*) in atria was observed in the Veh-treated group, whereas the *Npr2* and *Npr3* expression was significantly recovered in the SacVal-treated group; while the incremental recovery in their expression was not significant in the Val-treated group (Supplementary Figure S3C). In addition, pressure overload significantly suppressed the expression of *Mme* (encoding NEP) in atria with respect to both gene and protein expression levels, and SacVal recovered their expression significantly, whereas Val did not show a significant recovery (Figures 1F,G). In short, the gene expression profiles of NPs, their cognate receptors, and *Mme* in heart tissue were significantly altered in heart failure, and then SacVal restored those alterations as well as Val.

### 3.2 SacVal attenuated the susceptibility of AF in heart failure

**Figure 2:**
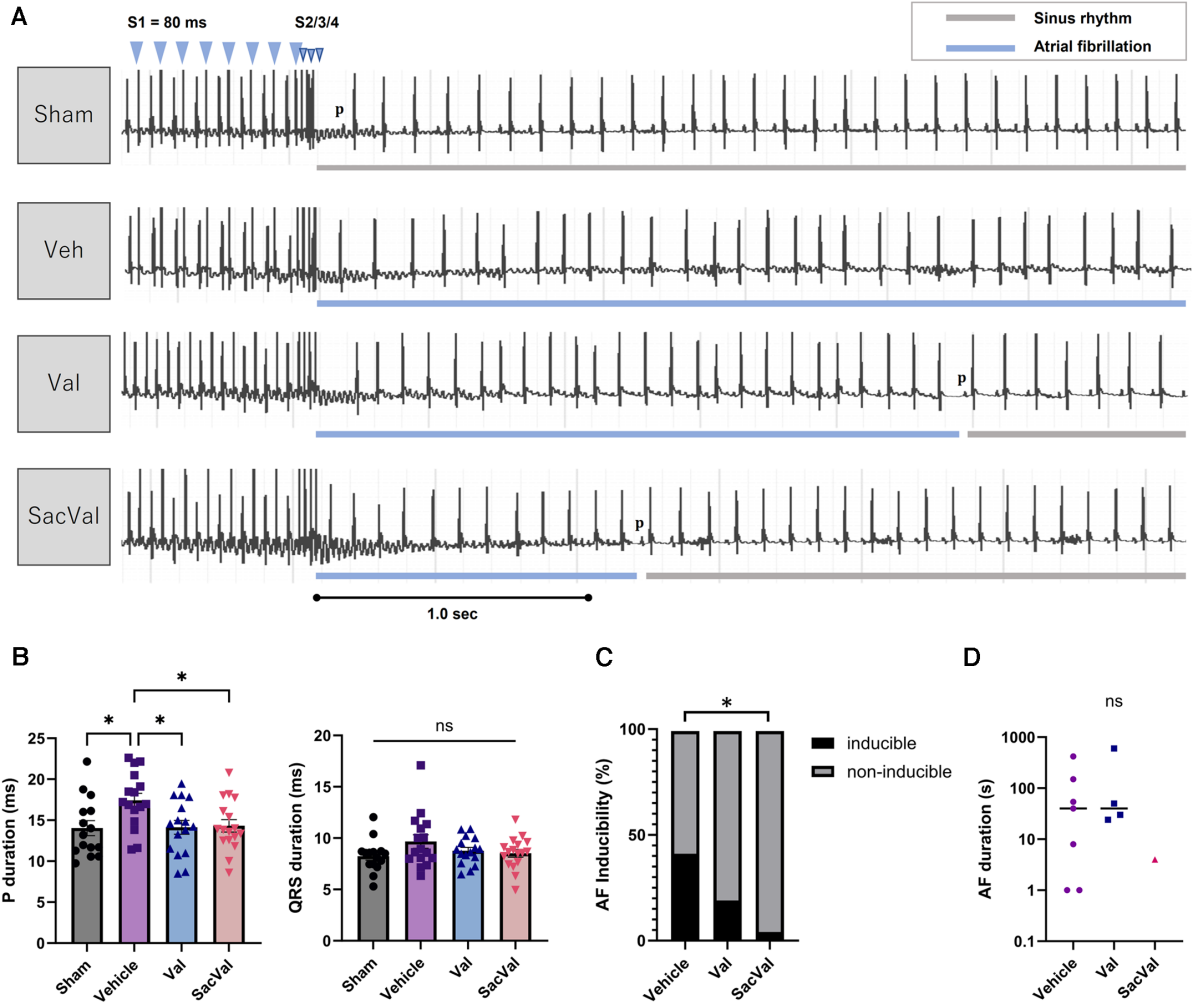
Electrophysiological assessment and AF inducibility in TAC mice. (**A**) Representative electrocardiograms of the induction and the termination of AF for each group. (**B**) *P*-wave duration (left) and QRS duration (right) (*n* = 15–17). Multiple comparison was performed using one-way ANOVA with Tuckey's post-hoc test. Error bars, SEM. *P < 0.05. The inducibility of AF (**C**) and the duration of induced AF (**D**) in each group (*n* = 17–22). Chi-square test adjusted with the Benjamini-Hochberg procedure was performed. *P < 0.05. Veh, vehicle; Val, valsartan; SacVal, sacubitril/valsartan; AF, atrial fibrillation; TAC, transverse aortic constriction.

To examine the influence of SacVal on atrial arrhythmogenicity, we assessed the inducibility of AF by programmed stimulation from the esophagus (Figure 2A). Surface ECG at baseline revealed that *P*-wave duration was increased in the Veh-treated group compared to that in the sham-treated group. Treatment with SacVal remarkably shortened the *P*-wave duration in comparison to the Veh-treated group, which was similar to that with Val (Figure 2B). Treatment with SacVal also tended to ameliorate the prolongation of QRS duration due to heart failure; however, these changes were not significant (Figure 2B). The programmed stimulation induced AF with a relatively high probability in the Veh-treated group and the occurrence of AF was significantly suppressed only in SacVal-treated group (40% vs. 4.5%), whereas the inducibility of AF in the Val-treated group was approximately 20% (Figure 2C). The duration of induced AF in the SacVal-treated group tended to be shorter than that in the Veh- or Val-treated groups (Figure 2D). These findings suggested that SacVal could reduce the susceptibility to AF in heart failure more than Val.

### 3.3 SacVal restored the impairment of atrial conduction and excitation property

**Figure 3:**
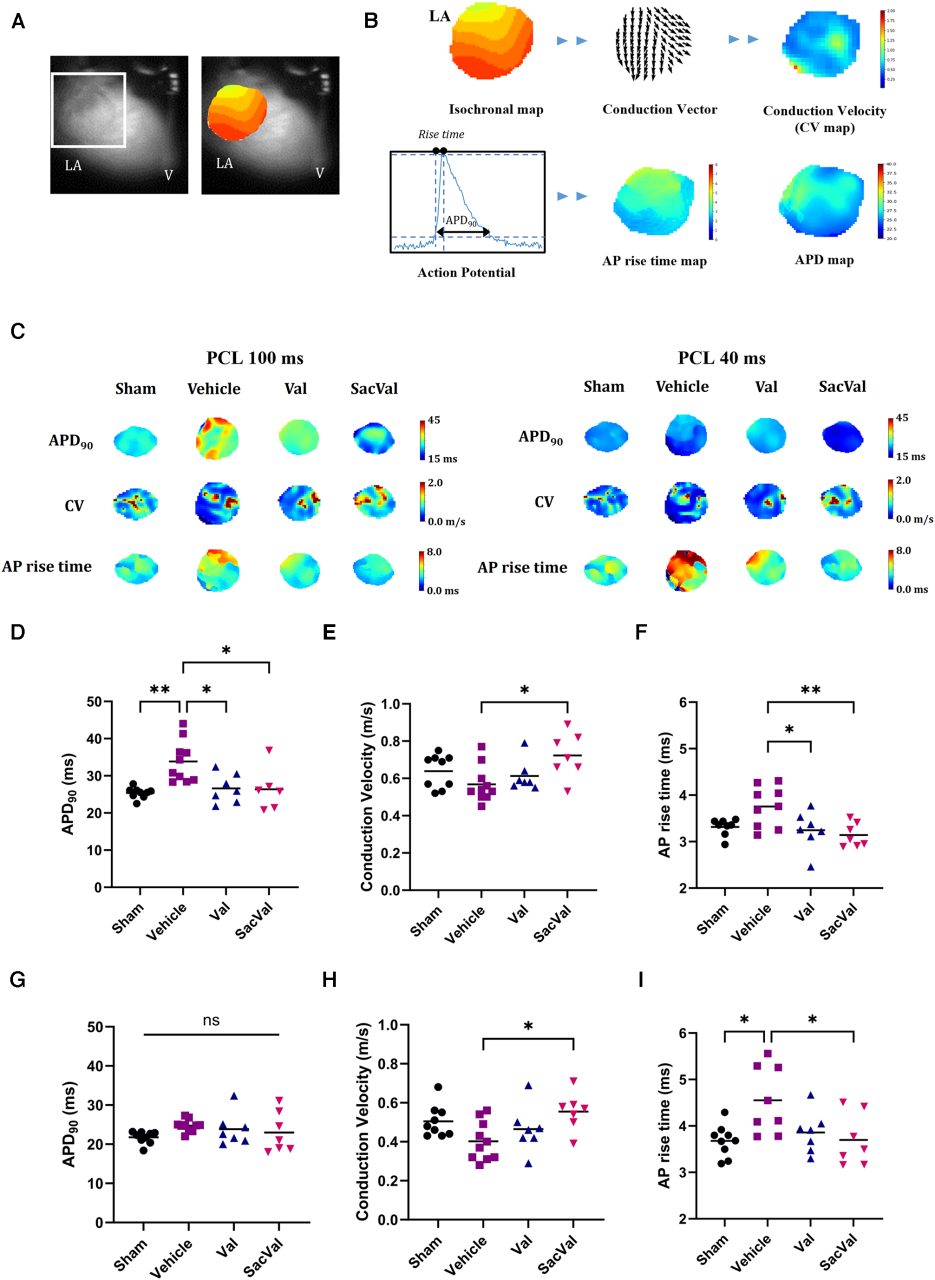
Optical mapping revealed that SacVal ameliorated the APD prolongation and the conduction disturbance. (**A**) Representative optical image and an atrial isochronal map overlaid. (**B**) A schema of signal processing to construct CV, APD and AP rise time map. (**C**) Representative electrophysiological maps of CV, APD_90_, and AP rise time at PCL 100 ms and 40 ms. APD_90_, CV, and AP rise time at PCL 100 ms (**D–F**) and PCL 40 ms (**G–I**) for each group (*n* = 7–10). Multiple comparison was performed using one-way ANOVA with Tuckey's post-hoc test. *P < 0.05; **P < 0.01. Val, valsartan; SacVal, sacubitril/valsartan; AP, action potential; APD, action potential duration; CV, conduction velocity; PCL, pacing cycle length.

**Figure 4:**
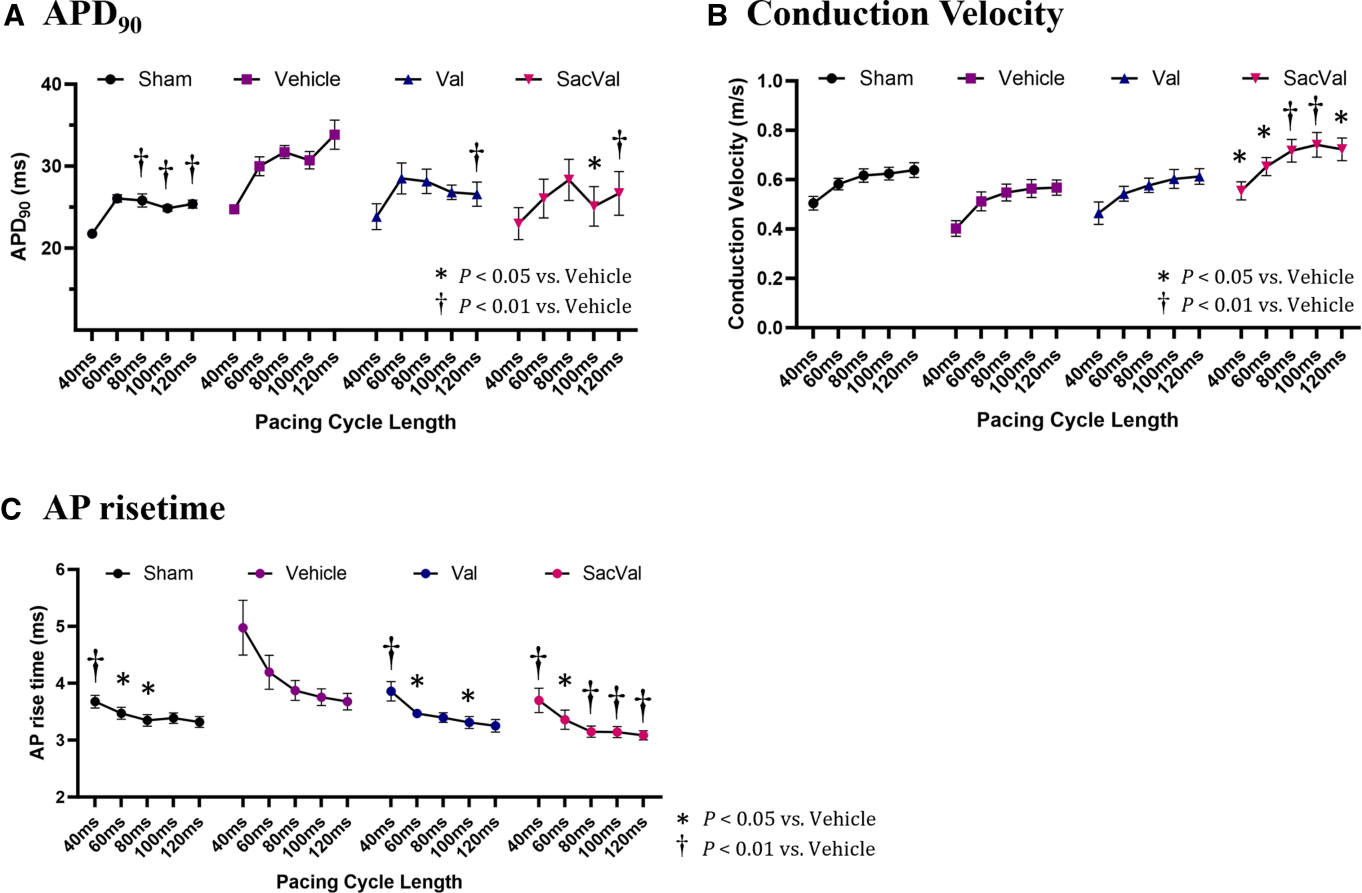
Restitution of APD, CV, and AP rise time. The restitution curves for (**A**) APD_90_, (**B**) CV, and (**C**) AP rise time. Multiple comparisons were performed using a two-way ANOVA with Tuckey's post-hoc test. Data are presented as mean ± SEM. *P < 0.05, ^†^*P* < 0.01 relative to vehicle. Val, valsartan; SacVal, sacubitril/valsartan; AP, action potential; APD, action potential duration; CV, conduction velocity.

To investigate the potential effects of SacVal treatment on atrial electrophysiological properties, the optical mapping with high spatiotemporal resolution was conducted. We processed the data obtained from atrial fluorescence into an isochronal map and an APD map, which were subsequently converted into a CV map and an AP rise time map (Figures 3A,B). These electrophysiological alterations were further assessed using a decremental PCL by 20 ms down to 40 ms. Representative maps for each group at PCL 100 ms and 40 ms are shown in Figure 3C. When compared between groups, APD maps revealed that the prolonged APD_90_ under heart failure was restored by both Val and SacVal significantly (Figure 3D). CV was reduced in the Veh-treated group, and SacVal-treatment reversed this reduction in CV, which was not observed in the Val-treated group (Figure 3E). This finding corresponds to the change in *P*-wave duration observed in the body-surface ECG. Similarly, the prolonged AP rise time observed in Veh-treated group got shortened significantly in SacVal-treated group as well as Val-treated group (Figure 3F). The difference in APD_90_ between the groups was diminished at a PCL of 40 ms (Figure 3G), whereas the significant restoration of CV and AP rise time was consistently observed even with a PCL of 40 ms only in the SacVal-treated group compared to the Veh-treated group (Figures 3H,I). The restitution curves of APD_90_, CV and AP rise time (Figures 4A–C) confirmed that SacVal improved those restitution in heart failure more effectively than Val. Taken together, SacVal might have a more favorable anti-arrhythmogenic effect than Val by preventing impaired atrial electrophysiological properties in heart failure.

### 3.4 SacVal restored the increased heterogeneity of the atrial electrophysiological properties

**Figure 5:**
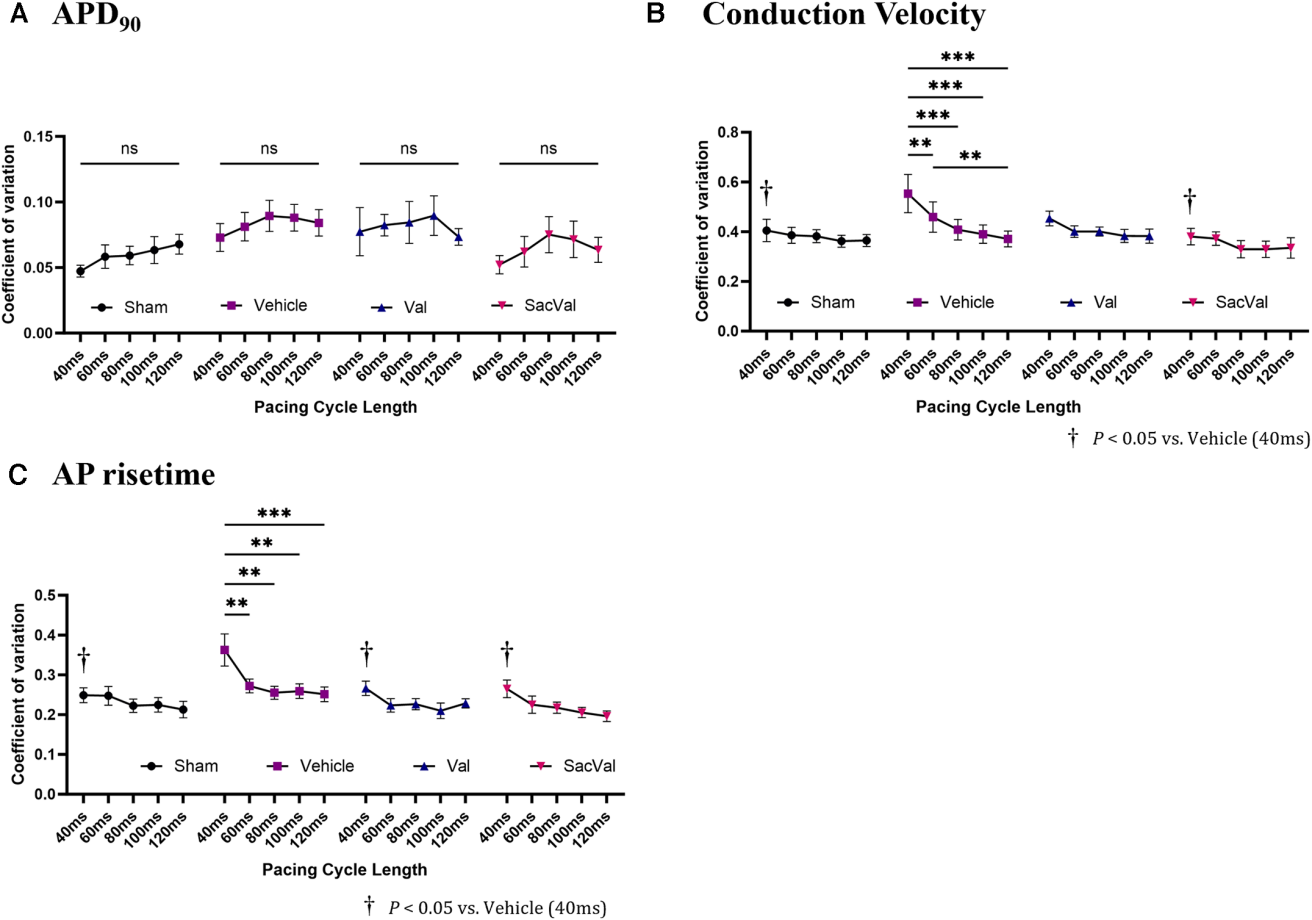
Sacval attenuated the enhanced heterogeneity of CV and AP rise time. The rate dependence of the coefficient of variation derived from (**A**) APD_90_, (**B**) CV, and (**C**) AP rise time maps (*n* = 6–8). Multiple comparisons within and between groups were performed using one-way and two-way ANOVA respectively, with Tuckey's post-hoc test. Data are presented as mean ± SEM. *P < 0.05; **P < 0.01; ***P < 0.001. ^†^*P* < 0.05 relative to vehicle at PCL 40 ms. Val, valsartan; SacVal, sacubitril/valsartan; AP, action potential; APD, action potential duration; CV, conduction velocity.

To further investigate the electrophysiological effects of SacVal, we evaluated the spatial heterogeneity of the APD_90_, CV, and AP rise time. The coefficient of variation (CoV) derived from each map was compared between the groups. The CoV of APD_90_ tended to be slightly smaller in all groups, with no significant differences as the PCL shortened (Figure 5A). In contrast, the CoV of the CV showed a tendency to increase with a shorter PCL in all groups. Notably, the CoV of the CV in the Veh-treated group significantly increased at PCL 40 ms, which was alleviated in the SacVal-treated group (Figure 5B), suggesting that SacVal could suppress the atrial heterogeneity of the CV in heart failure. Similarly, SacVal tended to suppress the increase in CoV of AP rise time (Figure 5C).

Regarding the electrophysiological alterations in the APD_90_, CV and AP rise time, we assessed the gene expression levels of ion channels, including connexins. Corresponding to the results of APD_90_, the downregulated gene expression of several subtypes of potassium ion channels, including *Kcnd2* and *Kchh2* in the Veh-treated group was restored in the SacVal-treated group, while SacVal did not restore the downregulated expression of *Kcnj2* (Supplementary Figure S4A). Additionally, *Cacna1c* and its encoding protein Cav1.2 did not show apparent differences between the groups (Supplementary Figures S4B,C). Meanwhile, despite the improvement of impaired atrial conduction by SacVal, only slight alterations in the expression of *Scn5a*, *Gja1*, *Gja5*, and their encoding proteins, Nav1.5, Cx43, Cx40, respectively were found, compared with Val (Supplementary Figures S4D,E, S5A–C); thus, these altered gene and encoding protein expressions could not explain the additional anti-arrhythmic effect of SacVal, and another mechanism could be contributing to it. Taken together, SacVal administration led to the less heterogeneity in the electrophysiological properties, which could contribute to improving the atrial arrhythmogenicity.

### 3.5 SacVal suppressed the atrial fibrosis in pressure overload

**Figure 6:**
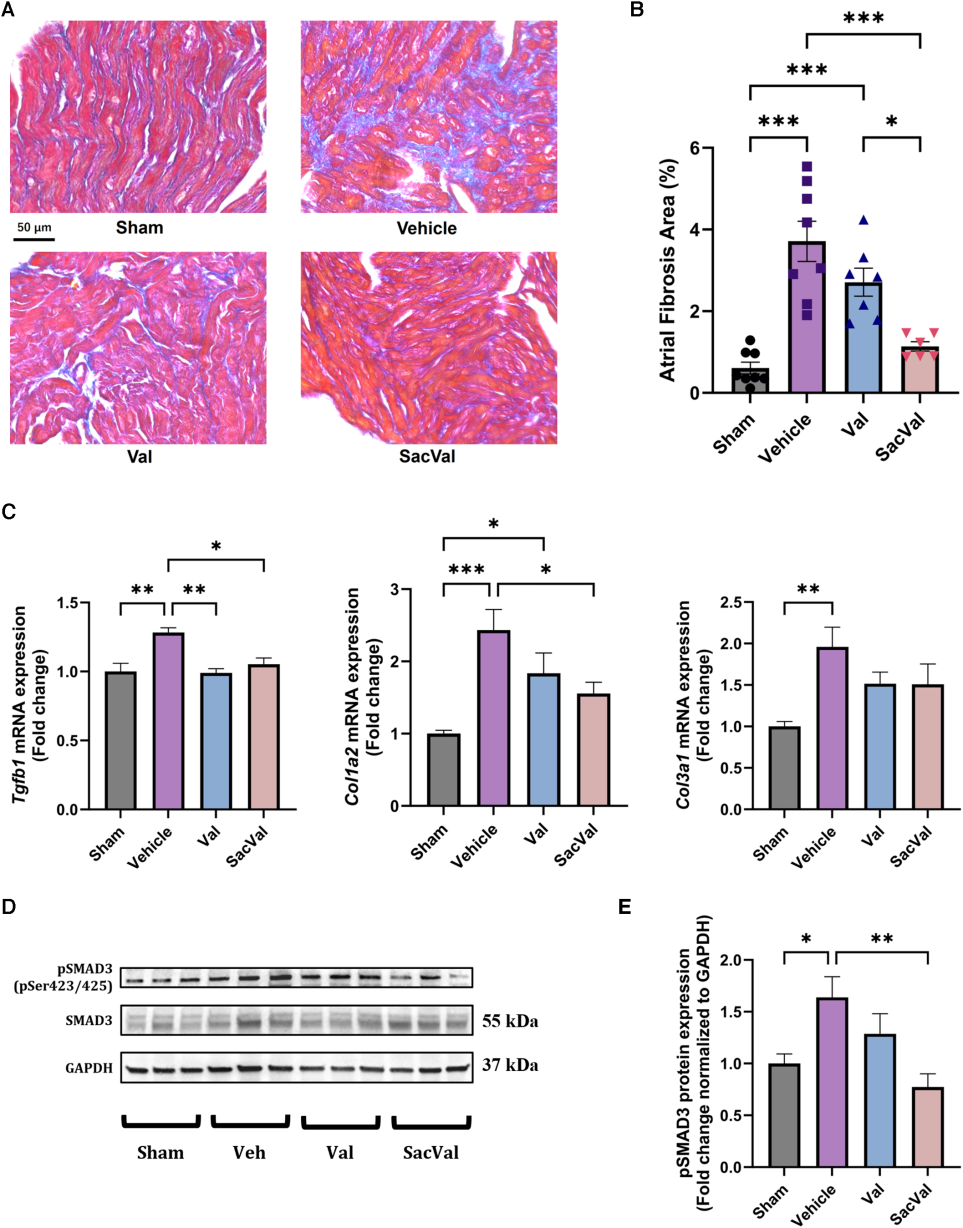
Sacval ameliorated the fibrotic change against murine cardiac remodeling. (**A**) Representative images of Masson's trichrome staining. Magnifying power of 40×. Scale bar = 50 *µ*m. (**B**) Quantification of atrial fibrosis area (%) (*n* = 6–8). (**C**) Relative gene expression regarding fibrosis: *Tgfb1*, *Col1a2*, and *Col3a1* (*n* = 6). (**D**) Western blotting images of Ser423/425-phosphorylated and non-phosphorylated SMAD3. (**E**) Relative protein expression of pSMAD3 to GAPDH (*n* = 6). Multiple comparison was performed using one-way ANOVA with Tuckey's post-hoc test. Error bars, SEM. *P < 0.05; **P < 0.01; ***P < 0.001. Val, valsartan; SacVal, sacubitril/valsartan.

Atrial fibrosis was examined to assess the underlying mechanism of the atrial electrophysiological effects of SacVal, particularly conduction heterogeneity. Histological assessment with Masson's trichrome staining showed that the deposition of collagen fibers in the atria significantly increased with heart failure, and SacVal significantly reduced the volume of atrial fibrosis compared with Veh and even Val (Figures 6A,B). The expression levels of the fibrosis-related genes, such as *Tgfb1, Col1a2* and *Col3a1*, in the SacVal-treated group were significantly suppressed to the same extent as those in the Val-treated group (Figure 6C). Similarly, the expressions of *Tnf*, *Il6* and *Il1b* were also assessed and found to be alleviated by SacVal and Val (Supplementary Figure S6). Furthermore, to investigate the signaling pathways related to fibrosis, the protein expression level of SMAD3, which is phosphorylated to activate the signaling cascade, was examined. Corresponding to the histological findings, the phosphorylated SMAD3 at Ser423/425 (pSMAD3) was elevated in the atria of TAC mice, whereas SacVal and not Val tended to attenuate the expression of pSMAD3 (Figures 6D,E). In summary, SacVal manifested the anti-fibrotic alterations in atria with heart failure, which could be more effective than Val, and might contribute to the beneficial effect of SacVal on atrial conduction properties.

### 3.6 Inhibition of neprilysin suppressed the activation of CFs *in vitro*

**Figure 7:**
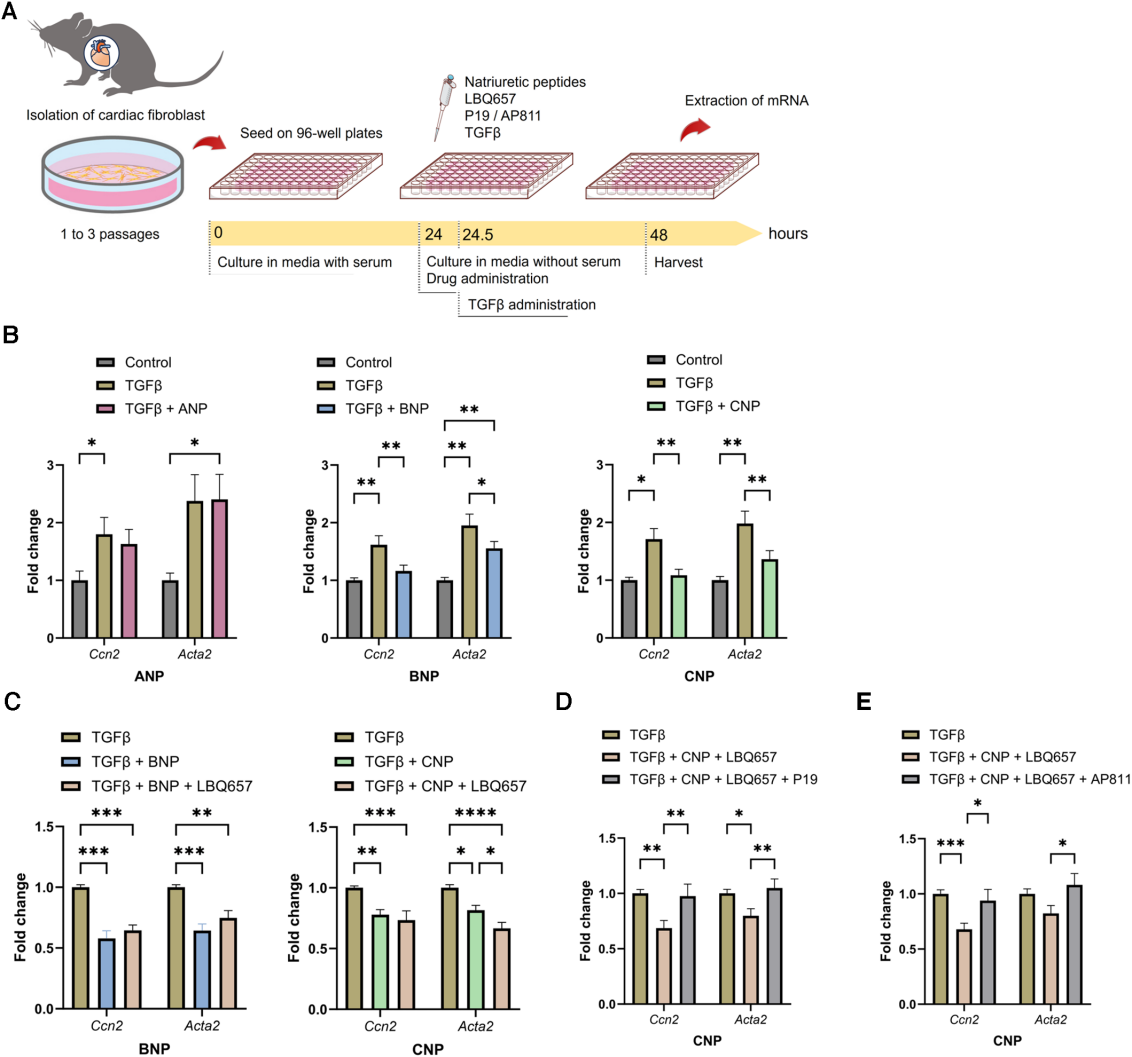
BNP and CNP suppressed the activation of primary adult cardiac fibroblasts. (**A**) The protocol of the *in vitro* assay. (**B**) Effects of ANP (left), BNP (middle) or CNP (right) (at 1.0 *µ*M each) against the pro-fibrotic alteration of *Ccn2* and *Acta2* induced by TGFβ (*n* = 5–8). (**C**) Additional effect of LBQ657 (50 *µ*M) on BNP (left) or CNP (right) (5.0 *µ*M each) (*n* = 6–7). (**D**) Antagonizing actions of P19 (1.0 *µ*M) on CNP (1.0 *µ*M) added with LBQ657 (10 *µ*M) (*n* = 6). (**E**) Effects of AP811 (1.0 *µ*M) on CNP (1.0 *µ*M) with LBQ657 (10 *µ*M) (*n* = 7). Multiple comparison was performed using one-way ANOVA with Tuckey's post-hoc test. Error bars, SEM. *P < 0.05; **P < 0.01; ***P < 0.001; ****P < 0.0001. ANP, atrial natriuretic peptide; BNP, B-type natriuretic peptide; CNP, C-type natriuretic peptide; TGFβ, transforming growth factor β.

According to previous *in vivo* and *in vitro* studies, the effects of NPs are assumed to be enhanced by NEP inhibitors (28, 29); hence, we examined the functions of ANP, BNP, and CNP against fibrosis *in vitro* using isolated CFs (Figure 7A). BNP and CNP conferred significant suppression of the gene expression of *Ccn2* and *Acta2* which indicate activation of CFs, after the application of exogenous TGFβ, whereas ANP did not bring any significant anti-fibrotic alterations (Figure 7B). Subsequently, we evaluated the anti-fibrotic effect of the NPs by adding LBQ657, a metabolite and an active form of sacubitril. Intriguingly, the addition of LBQ657 to CNP, but not to BNP, was associated with a significant enhancement of the anti-fibrotic effect, suggesting that sacubitril could enhance the anti-fibrotic effect of CNP rather than BNP on CFs, at least *in vitro* (Figure 7C). Similar trends were observed for the expressions of *Col1a2* (Supplementary Figure S7). To confirm that the effects of LBQ657 were attributed to the prevention of CNP degradation, we evaluated whether the additional effect of LBQ657 was antagonized by the NPR-B inhibitor, P19. We found that the existence of P19 inhibited the anti-fibrotic effects of CNP (Figure 7D). Furthermore, the anti-fibrotic effect of CNP was similarly diminished by the addition of the NPR-C inhibitor, AP811 (Figure 7E). These results indicate that the additional anti-fibrotic effect when SacVal was added was partly mediated by CNP via NPR-B and NPR-C.

## 4 Discussions

In the present study, we clearly described, for the first time, the additional anti-arrhythmic effects of SacVal *in vivo* by utilizing the precise assessment of atrial electrophysiological properties by detailed optical mapping. Previous reports have shown that SacVal has an anti-fibrotic effect on murine atria under several pathological conditions (19, 30); however, its effect on atrial arrhythmogenicity and the underlying electrophysiological changes remain elusive. Our investigation for the rate dependence of electrophysiological properties showed that SacVal can prevent not only the reduction of conduction velocity, but also the increase in its heterogeneity even under short PCL. Therefore, our findings provide novel evidence that SacVal suppresses atrial arrhythmogenicity *in vivo* by attenuating electrophysiological heterogeneity and fibrosis in heart failure. Moreover, we demonstrated that the neprilysin inhibitor amplified the anti-fibrotic function of CNP for isolated CFs using *in vitro* assay. Although the contribution of CNP to heart failure, compared with ANP and BNP, is controversial (28, 29, 31, 32), our *in vitro* results indicated that CNP is a possible mediator of neprilysin inhibition in heart failure.

In this study, impaired atrial conduction properties in heart failure were significantly improved by the amelioration of atrial fibrosis by SacVal. SacVal also restored the alteration in the AP rise time induced by TAC; however, the expression of *Scn5a* and *Kcnj2* was not restored. Cardiac remodeling involves the activation and proliferation of cardiac fibroblasts, leading to increased deposition of the extracellular matrix and impeding the propagation of excitation within the cardiac tissue (33, 34). In addition to insulator obstacles due to the deposition of collagen fibers, previous studies have demonstrated direct electrical couplings between cardiomyocytes and cardiac myofibroblasts through connexins (35–37). This coupling enables the myofibroblasts to act as current sinks or poor conductors (35). Thus, it can promote the reduction of CV via elevation of resting membrane potential in cardiomyocytes, which can eventually lead to the inactivation of sodium channels and the prolonged AP rise time (34, 38, 39). In other words, the alteration in the AP rise time observed in this study might be affected by cardiomyocyte-fibroblast coupling.

Regarding regional electrophysiological heterogeneity, our findings demonstrated that rapid atrial activation with a shorter cycle length induced incremental heterogeneity of CV in TAC mice which is known to be closely correlated with AF (40, 41). Previous reports indicated that increased tissue fibrosis, which contributes to increased regional conduction heterogeneity, is associated with the initiation and perpetuation of AF (42–45). Therefore, SacVal treatment can be considered to improve atrial arrhythmogenicity not only by restoring decreased CV, but also by ameliorating the heterogeneity of CV in this study. However, whether fibrotic dispersion coincides with electrophysiological heterogeneity remains controversial (43). Thus, further investigation of the attributes of conduction heterogeneity is required.

Our *in vivo* and *in vitro* investigation of SacVal implied that SacVal had additional anti-fibrotic effects to Val. In the *in vitro* assay, the anti-fibrotic function of CNP was potentiated by the addition of LBQ657, which implied that the additional anti-fibrotic effect of SacVal was partly derived from the augmentation of CNP function by sacubitril. A previous study reported that BNP is considerably more resistant to degradation by NEP than ANP and CNP (46, 47), therefore, it is possible that the anti-fibrotic effect of BNP was not enhanced by LBQ657 in this study. In contrast, CNP is the preferred substrate for NEP (46, 47). CNP is considered a ubiquitous autocrine/paracrine regulator; thus, its serum concentration is much lower than that of ANP and BNP (29, 48). Despite the lack of an increase in circulating CNP, the local tissue concentration of CNP in atria is elevated under heart failure (31), which corresponds to our result that the expression level of CNP in atria increased in heart failure. To date, CNP has come to be regarded as a major heart-protective NP in a failed heart and has an anti-remodeling effect on the heart, such as anti-fibrosis, anti-inflammation, and microcirculatory vasodilation (49, 50). These reports support our findings on CNP.

This study also suggests that CNP affects CFs through both NPR-B and NPR-C. According to a previous study in transgenic mice, over-expression of the dominant negative form of NPR-B in cardiomyocytes results in accelerated developments of cardiac hypertrophy, fibrosis, and contractile dysfunction (51). Furthermore, CNP has been reported to exert cardioprotective effects via NPR-C, which is regarded as a clearance receptor of NPs, and NPR-C^−/−^ mice exhibit significantly worse cardiac phenotypes, including fibrotic proliferation, in response to pressure overload (52). Notably, fibroblast-specific CNP^−/−^ mice exhibit exacerbated collagen deposition and worsened cardiac function when exposed to pressure overload (52). In summary, the additional anti-fibrotic effect of SacVal observed in this study might be mediated by CNP via NPR-B and NPR-C.

This study had some limitations. First, the effects of Val alone, which showed sufficient effectiveness, made it difficult to detect differences when compared with those of SacVal. However, even in such situations, SacVal has demonstrated significant additional anti-arrhythmic effects. The advantages of SacVal may be demonstrated more clearly using other experimental conditions or mouse models of cardiac diseases. Second, cardiac fibroblasts were isolated from both atria and ventricles for *in vitro* assays, so results from the cells are not specific to atrial remodeling. Moreover, the cardiac fibroblasts were not from the TAC-operated mice, but were treated with TGFβ to activate them, so the *in vitro* experiments do not exactly mimic atrial remodeling with the stress induced by TAC *in vivo*, and using atrial fibroblasts isolated from TAC-operated mice would be the optimal option. Third, the present study lacked an *in vivo* assessment of the effect of NPR-B or NPR-C blockers on SacVal as they were unavailable; thus, we could not determine the extent to which CNP contributes to atrial fibrosis *in vivo*. Further study using mutant mice with knockouts of these proteins would be helpful. Fourth, SacVal improved not only the atrial electrophysiological properties but also the ventricular function in this study. Therefore, it is difficult to rule out the possibility that SacVal may have demonstrated its effects as a secondary effect on the improvement of heart failure. Lastly, our electrophysiological assessment using optical mapping focused only on the LA and not on the entire atrium. Electrophysiological differences between the LA and right atrium or pulmonary veins could also contribute to atrial arrhythmogenicity. The electrical interactions between LA and other regions require further investigation.

Taken together, our results demonstrate that SacVal improves conduction disturbance through the inhibition of fibrosis in murine atria with pressure overload, which may be attributed to the enhanced function of CNP.

## Data Availability

The original contributions presented in the study are included in the article/Supplementary Material, further inquiries can be directed to the corresponding author.
