# Supplementary material for: Sacubitril/valsartan attenuates atrial conduction disturbance and electrophysiological heterogeneity with ameliorating fibrosis in mice
Source: Front Cardiovasc Med. 2024 Jan 19;11:1341601. doi: 10.3389/fcvm.2024.1341601 (PMC10834649; doi:10.3389/fcvm.2024.1341601)
Supplement: Supplementary file 1 [file Datasheet1.pdf]

Supplementary Material

1 Supplementary Figures and Tables

1.1 Supplementary Tables

Supplementary Table S1

Sequences of primers used for quantitative PCR

Gene	Forward	Reverse
Nppa	GAAGATGCCGGTAGAAGATGAGGT	ACTCTGGGCTCCAATCCTGTCAAT
Nppb	GCCAGTCTCCAGAGCAATTCA	TGTTCTTTTGTGAGGCCTTGG
Nppc	CTCTACTTTGGCTGGTTCCTGAC	GCTTCTCCTTGGGTTCATCTCC
Npr1	TGGGCGAGGCTCCAATTATG	CTGCACATCCCGCATATGTT
Npr2	CTTCAATTGGACAGCTCGGG	GCCGCAGATATACACAATGCG
Npr3	ATGATGCTCGCTCTGTTTCG	ACATGATCACCACTCGCTC
Mme	CTCTCTGTGCTTGTCTTGCTC	GACGTTGCGTTTCAACCAGC
Tgfb1	GTGCGGCAGCTGTACATTGACTTT	TGTGTTGGTTGTAGAGGGCAAGGA
Col1a2	CCCCGGGACTCCTGGACTT	GCTCCGACACGCCCTCTCTC
Col3a1	GGAAAGGATGGAGAGTCAGGAA	CATTGCGTCCATCAAAGCCT
Il6	ACAACCACGGCCTTCCCTACTT	CACGATTTCCCAGAGAACATGTG
Il1b	CTGGTGTGTGACGTTCCCATTA	CCGACAGCACGAGGCTTT
Tnf	ACCCTCACACTCAGATCATCTTC	TGGTGGTTTGCTACGACGT
Ccn2	CTGCGAGGAGTGGGTGTG	ATGTGTCTTCCAGTCGGTAGG
Acta2	ACTCTCTTCCAGCCATCTTTC	ATAGGTGGTTTCGTGGATGC
Cacna1c	TGCTGTACTGGATGCAAGACGCTA	GCATGCTCATGTTTCGAGGCTTGT
Scn5a	AGAAGACGGTCCCAGAGCATTGAA	ATGACCAGGAGCTGAGGTTCCAAA
Kcnd2	GCCGCAGCACCTAGTCGTT	CACCACGTCGATGATACTCATGA
Kcnj2	CCTTTGTAGTGCCAGAGACTTAG	TCCTCTTCCTCTTTGCTTGTTAG
Kcnq1	CAAAGACCGTGGCAGTAAC	CCTTCATTGCTGGCTACAAC
Kcnh5	TTTCCAGGAACCTCACTCTCAC	CTCCTCCTCCTTCTTCACATCA
Kcnj5	GAGTTCGAAGTTGTGGTCATA	GCACCTCTGTATCCATGTAAG
Kcnj11	CAAGATGCACTTCAGGCAAA	CCAGGCTGAACTTCCCAATA
Gja1	AACAGTCTGCCTTTCGCTGT	GGGCACAGACACGAATATGA
Gja5	ATTCTGATCCGCACCACCAT	CATGCAGGGTATCCAGGAAGA
Myh6	GGAAGAGTGAGCGGCGCATCAAGG	CTGCTGGAGAGGTTATTCCTCG
Myh7	GCCAACACCAACCTGTCCAAGTTC	TGCAAAGGCTCCAGGTCTGAGGGC

Gapdh	TGTGATGGGTGTGAACCACGAGAA	GAGCCCTTCCACAATGCCAAAGTT

Supplementary Table S2

Antibodies used for Western Blotting

Primary antibodies

Antibody	Host animal	Company	Catalog number	Dilution
SMAD3	Mouse	Proteintech Inc.	66516-1-lg	1:1000
SMAD3 pSer423/425	Rabbit	GeneTex	GTX129841	1:500
NPR-B	Rabbit	Bioss Inc.	bs-2348R	1:500
NPR-C	Rabbit	GeneTex	GTX64458	1:1000
Neprilysin	Mouse	R&D Systems	AF1126-SP	1:500
ANP	Goat	Santa Cruz Biotechnology	sc-18811	1:500
CNP	Rabbit	MyBioSource	MBS2026757	1:500
Cx43	Rabbit	Santa Cruz Biotechnology	sc-9059	1:500
Cx43 pSer368	Rabbit	Novus Biologicals	NB100-81867	1:500
Cx40	Goat	Santa Cruz Biotechnology	sc-20466	1:500
Cav1.2	Rabbit	Santa Cruz Biotechnology	sc-25686	1:500
Nav1.5	Rabbit	Proteintech Inc.	23016-1-AP	1:500
GAPDH	Mouse	Santa Cruz Biotechnology	sc-32233	1:500

Secondary antibodies

Primary Antibody	Host animal	Company	Catalog number	Dilution
Mouse	Goat	abcam	Ab205719	1:10000
Rabbit	Goat	Dako	P0448	1:10000
Goat	Rabbit	Dako	P0449	1:10000

1.2 Supplementary Figures

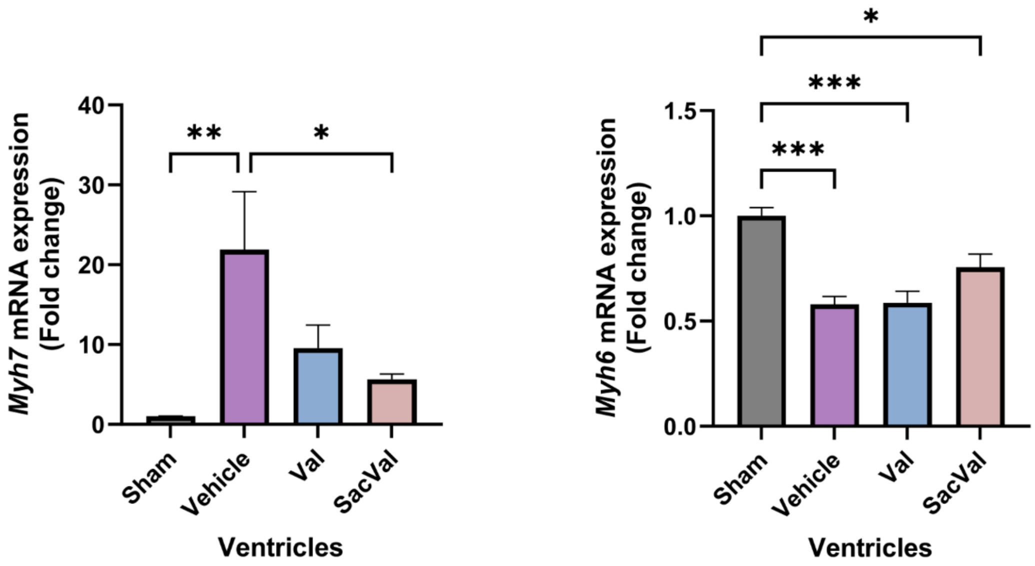

Supplementary Figure S1. Gene expression of hypertrophy-related genes in ventricles: *Myh7*, *Myh6*.
n = 6. Multiple comparison was performed using one-way ANOVA with Tuckey's post-hoc test. Error bars, SEM. **P* < 0.05; ***P* < 0.01; ****P* < 0.001. Val: valsartan, SacVal: sacubitril/valsartan**.**

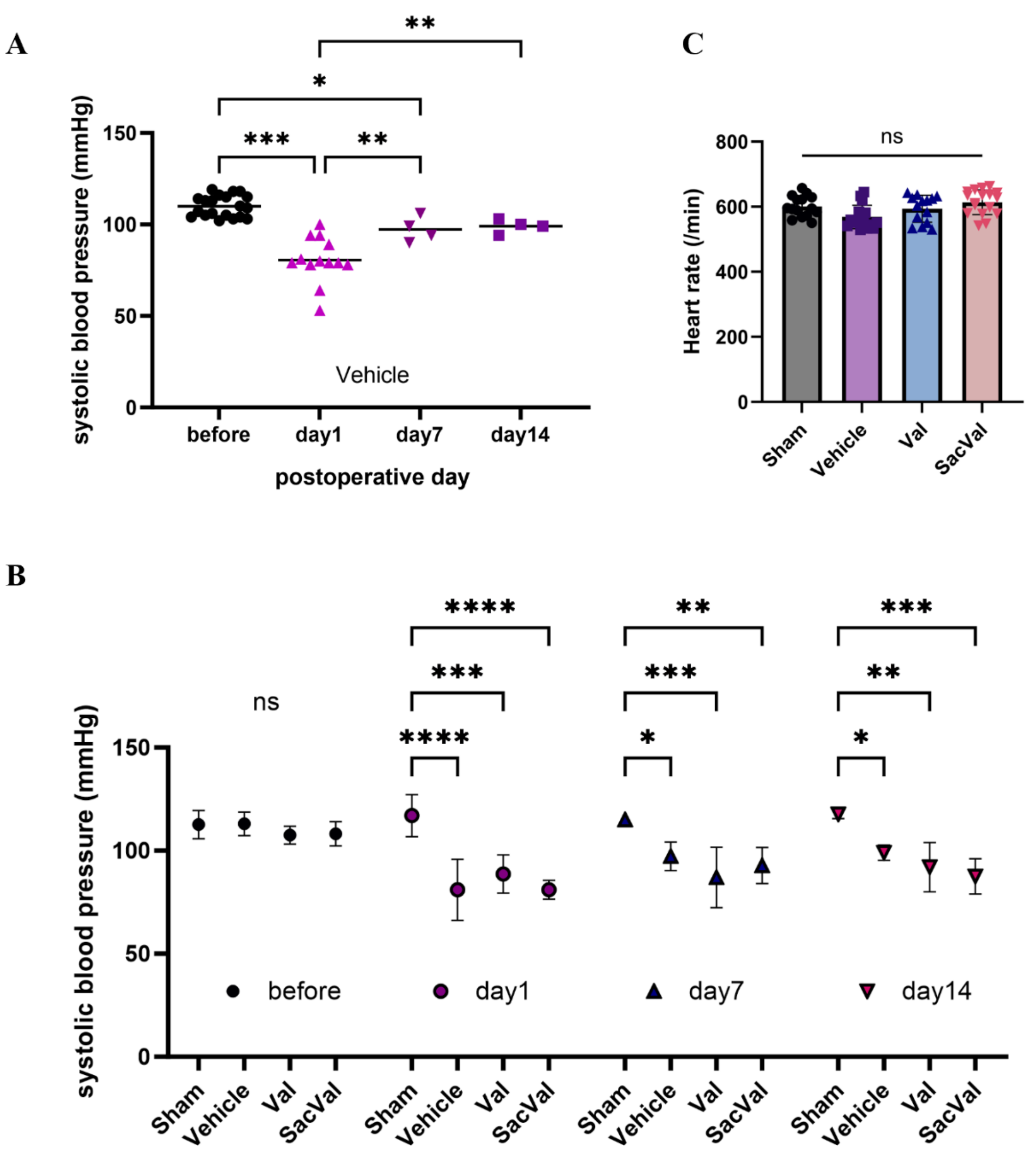

Supplementary Figure S2. Blood pressure and heart rate.

(A) Systolic blood pressure at different time points over the experimental course, before (n = 17) and postoperative day 1 (n = 13), 7 (n = 4), 14 (n = 4) after TAC surgery in the vehicle-treated group. (B) Comparisons of blood pressure between the groups before and on days 1, 7, 14 after surgery (n = 4 each group). (C) Heart rate on postoperative day 14 for each group. Multiple comparisons were performed using one-way ANOVA with Tuckey's post-hoc test. Data are presented as mean ± SEM. **P* < 0.05; ***P* < 0.01; ****P* < 0.001; *****P* < 0.0001. TAC: transverse aortic constriction, Val: valsartan, SacVal: sacubitril/valsartan.

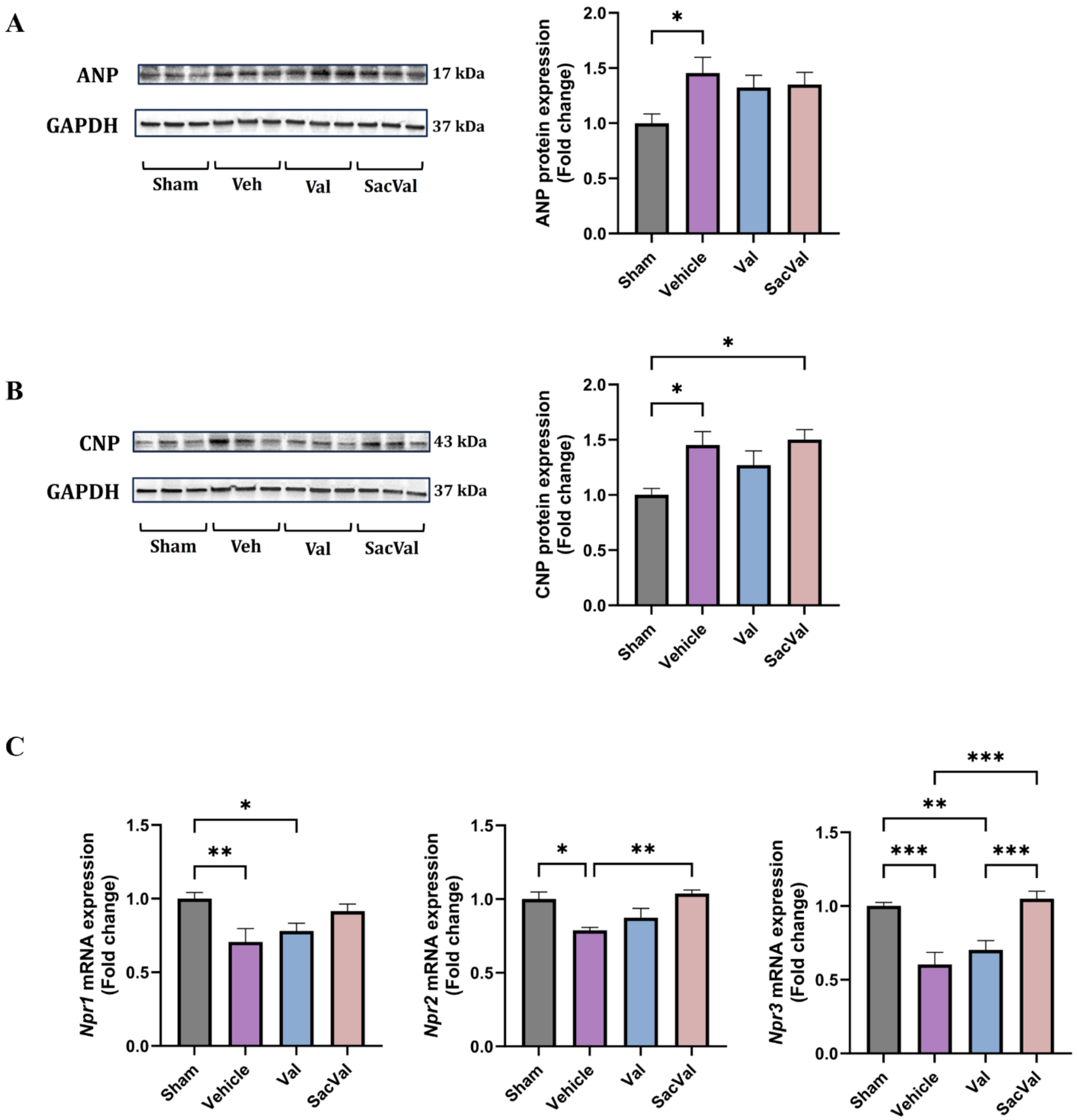

Supplementary Figure S3. Protein expression of ANP and CNP, and gene expressions of cognate receptors of natriuretic peptides.

(A, B) Atrial protein expression levels of ANP and CNP. (C) Atrial mRNA gene expression levels for the cognate receptors of natriuretic peptides; *Npr1*, *Npr2,* and *Npr3*. Multiple comparison was performed using one-way ANOVA with Tuckey's post-hoc test (n = 6). Error bars, SEM. *$P < 0.05$; **$P < 0.01$; ***$P < 0.001$. ANP: atrial natriuretic peptide, CNP: C-type natriuretic peptide, Val: valsartan, SacVal: sacubitril/valsartan**.**

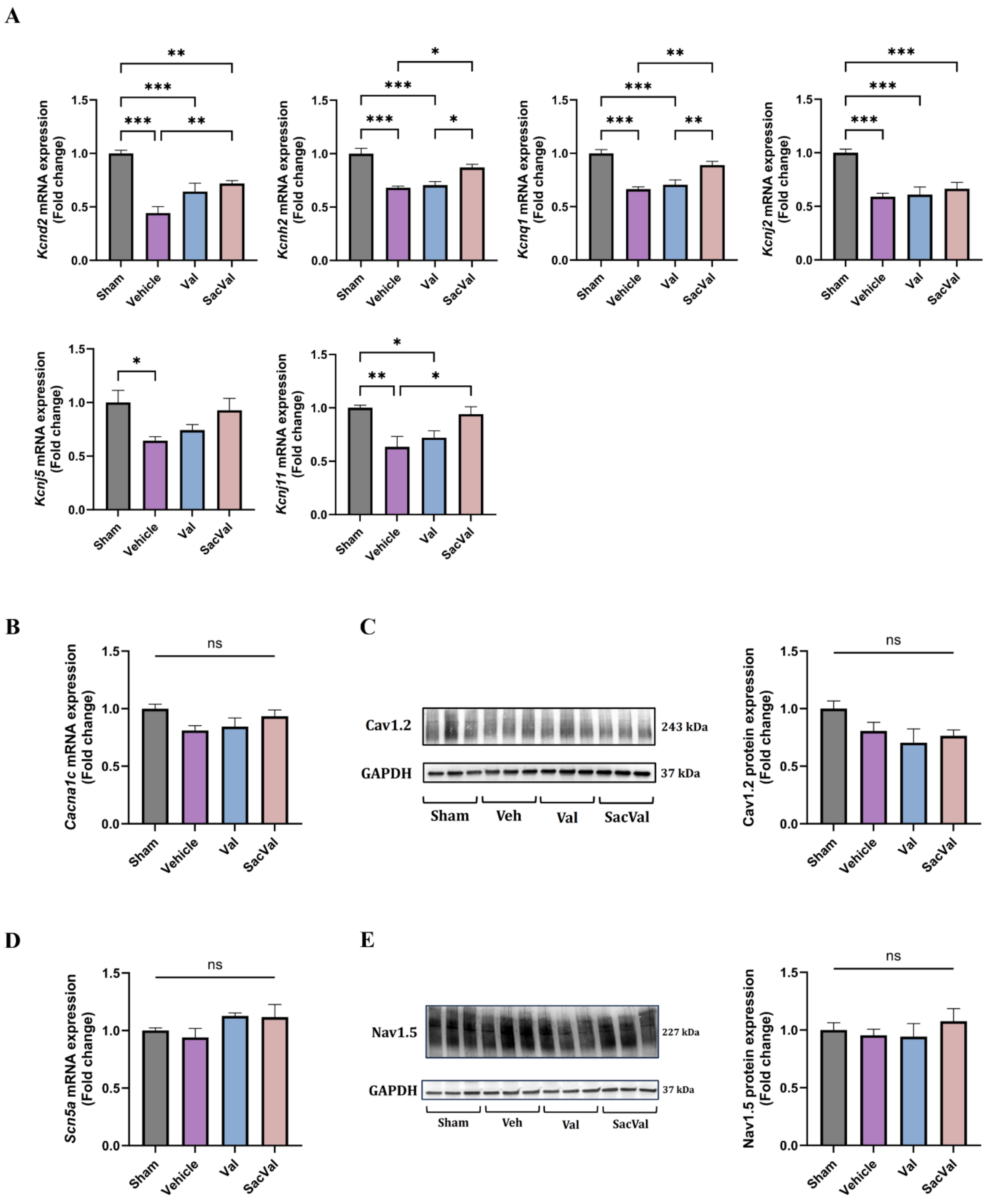

Supplementary Figure S4. Atrial mRNA gene and protein expression levels of ion channels.
(A) Potassium channels; *Kcnd2, Kcnh2, Kcnq1, Kcnj2, Kcnj5, Kcnj11*. (B, C) *Cacna1c* and encoding Cav1.2. (D, E) *Scn5a* and encoding Nav1.5 (n = 6). Multiple comparison was performed using one-way ANOVA with Tuckey's post-hoc test. Error bars, SEM. *$P < 0.05$; **$P < 0.01$; ***$P < 0.001$. Val: valsartan, SacVal: sacubitril/valsartan**.**

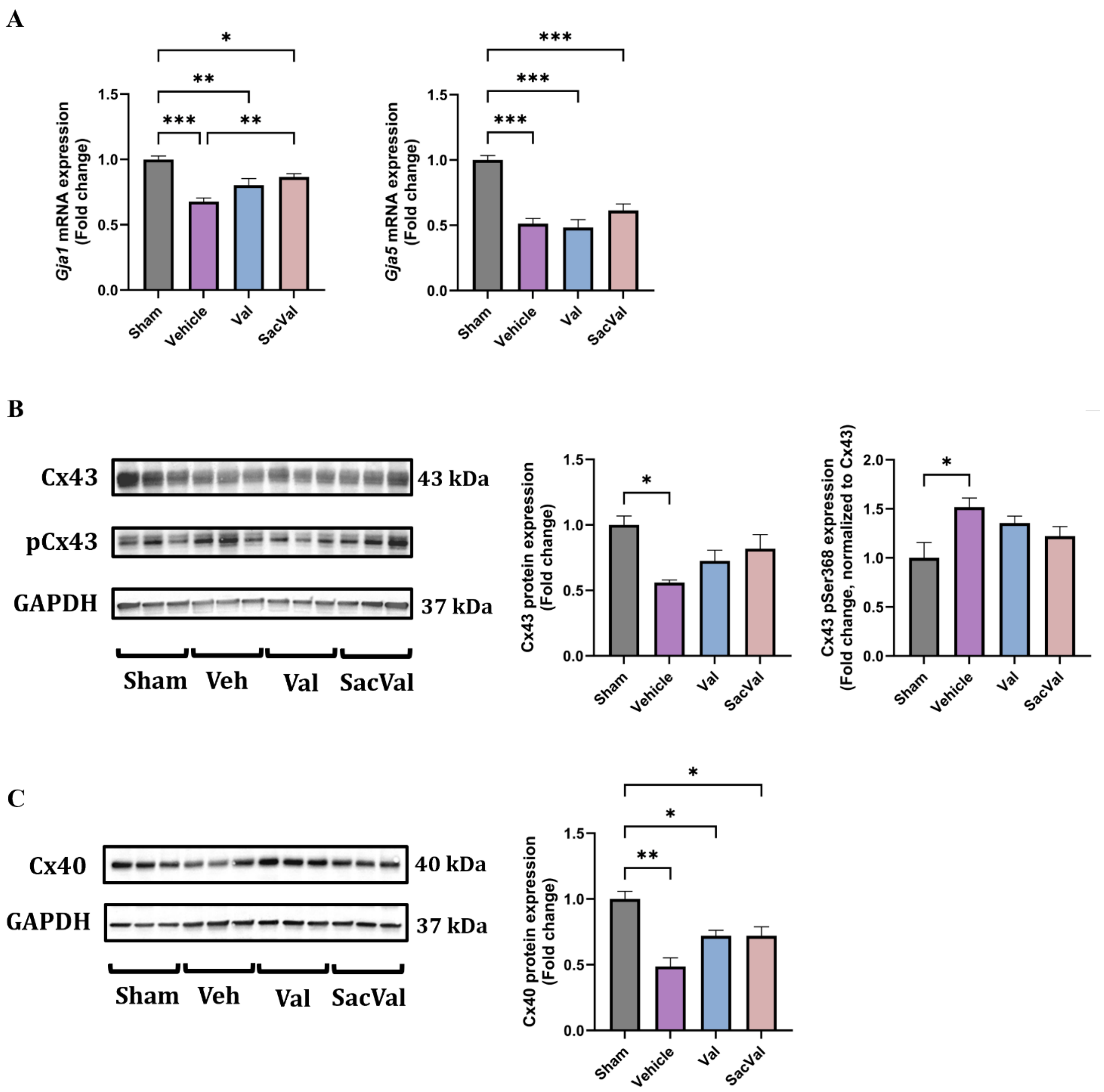

Supplementary Figure S5. Gene and protein expression of *Gja1* (Cx43) and *Gja5* (Cx40).
(A) *Gja1* and *Gja5* gene expression in atria (n = 6). (B) Atrial protein expression of Cx43 and serine368-phosphorylated Cx43 (pCx43) (n = 3). (C) Atrial Cx40 protein expression (n = 3). Multiple comparison was performed using one-way ANOVA with Tuckey's post-hoc test. Error bars, SEM. **P* < 0.05; ***P* < 0.01; ****P* < 0.001. Val: valsartan, SacVal: sacubitril/valsartan**.**

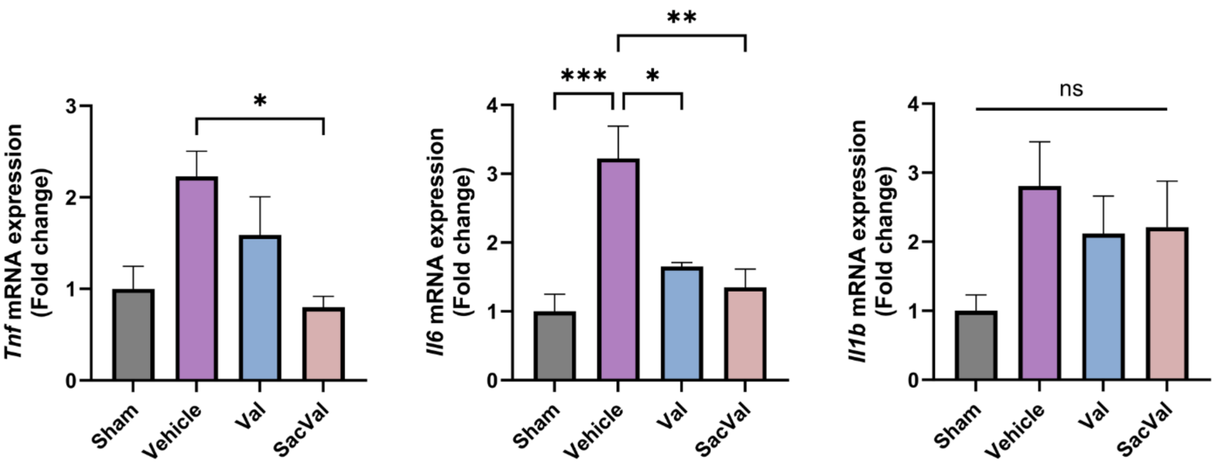

Supplementary Figure S6. Gene expression of inflammation-related genes: *Tnf*, *Il6*, *Il1b*
n = 4. Multiple comparison was performed using one-way ANOVA with Tuckey's post-hoc test. Error bars, SEM. **P* < 0.05; ***P* < 0.01; ****P* < 0.001. Val: valsartan, SacVal: sacubitril/valsartan**.**

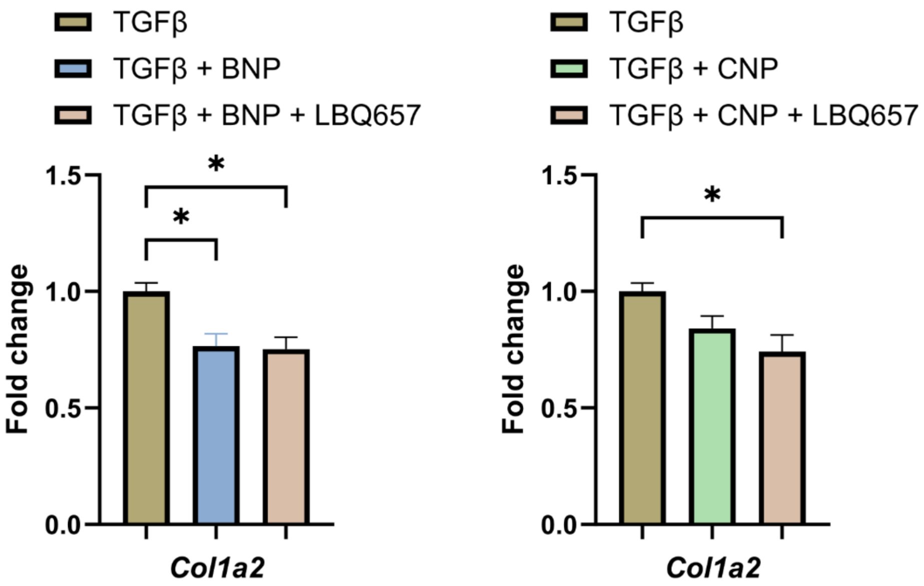

Supplementary Figure S7. Alterations of *Col1a2* mRNA expressions for cardiac fibroblasts in response to LBQ657 applied with BNP or CNP

n = 6. Multiple comparison was performed using one-way ANOVA with Tuckey's post-hoc test. Error bars, SEM. *$P < 0.05$. BNP: B-type natriuretic peptide, CNP: C-type natriuretic peptide, TGFβ: transforming growth factor β.
